# Relational Attributes Associated with Treatment Adherence: A Scoping Review

**DOI:** 10.2147/PPA.S604683

**Published:** 2026-07-29

**Authors:** Yi Jiao Angelina Tian, Emma Herger, Aoife Milford, Stephen Milford

**Affiliations:** 1 Institute for Biomedical Ethics, University of Basel, Basel, Switzerland; 2 Faculty of Theology, North-West University, Potchefstroom, South Africa

**Keywords:** provider-patient relationship, treatment adherence, empathy, trust, continuity of care

## Abstract

**Introduction:**

Treatment adherence improves health outcomes, reduces mortality, and saves healthcare costs. Since Hippocrates, patients’ adherence has been a concern in health management and its factors found to be complex. Existing research has pointed to positive impacts of strong provider-patient relationships (PPRs) on health outcomes and adherence. This scoping review explores the specific relational attributes in existing literature within PPRs that directly affect adherence and draws out gaps for future research.

**Methods:**

The databases CINAHL, Embase, and PubMed were searched for peer-reviewed articles published between 01.01.2015 and 07.01.2025 in English. Qualitative, quantitative, RCTs, and other empirical study designs were included. Included papers are those that report on any attribute(s) of the PPR that must be directly linked to treatment adherence.

**Results:**

Across 48 empirical studies included, we found 6 relational aspects of the PPR that were critical to adherence. Communication was cited to be the most important factor, particularly the content, communicative style, and frequency of information given to patients. Additional factors were also found, such as shared decision-making to empower patients in their own care, trust in the provider, provider’s empathy towards the patient, the continuity of care in the duration of the relationship, as well as the lack of discrepancies between provider and patient perspectives towards treatment adherence. Nevertheless, most articles lacked definitions and standardized measurements of these attributes, which we believe is a major gap in the research on PPR and its associated implications for healthcare.

**Discussion:**

The PPR plays a complex but important role in adherence. More efforts should be made to clarify and standardize the attributes in PPRs in order to better understand *how* and *by which mechanisms* adherence may be promoted through the relational dimension of care. Our review also reflects upon these results with overall societal shifts such as digitalization, the movement from compliance to patient-centred adherence, as well as specialization of medicine away from relational care. Findings could translate practically to improve patient adherence by delineating clear and well-defined steps for providers towards the frequency and quality of engagement in clinical encounters towards patients.

## Introduction

The phenomenon of adherence has been a central concern for healthcare ever since 400 B.C. when Hippocrates noted how patients declined to take their prescribed medication and complained about their treatment effectiveness.^1^ The term “adherence” generally refers to the extent to which a patient agrees, participates in, or follows a treatment regimen recommended by a healthcare provider.^2^,^3^ Such treatment regimens could include taking medication or making lifestyle changes (eg, healthy eating, exercise, or refraining from alcohol or tobacco). Patient adherence may be evaluated using numerous methods. For example, through direct observation of treatment effectiveness, counting medicinal dosage possession, detecting drug formulations in physiology, or through patient self-reports.^4^

A systematic review by Walsh et al^5^ found that “good adherence was associated with a 21% reduction in long-term mortality risk”, as well as decreasing the risks of hospitalization and improving clinical outcomes^5^ (pp 2464). This applies even when treatment is a placebo,^6–8^ where an early study found that cancer patients with excellent compliance responded best with the least incidence of fever or infection, regardless of whether they were randomized to the placebo or treatment group.^8^ The effects of adherence and health outcomes are especially relevant in the management of chronic illnesses. For example, the treatment of antiretroviral therapy for HIV/AIDS depends heavily on patients reaching a minimal threshold of adherence (~95%) so as to ensure sufficient therapeutic concentration of medicine required for the suppression of viral replication.^9^,^10^ Thus, ensuring high adherence in HIV/AIDS is directly related to long-term health outcomes, quality of life, and improved immunity that allows patients to live a near-normal life.^11^,^12^ In addition, adherence is directly linked to a reduction of healthcare costs.^13^ For example, it has been shown that non-adherent diabetes patients incur significantly greater pharmacy costs as well as inpatient and outpatient care costs.^14^ Similar patterns have been identified in patients suffering from heart failure or myocardial infraction, whereby adherence was associated with lower risks of rehospitalization and lower healthcare costs generally.^13^

Despite its importance, adherence rates vary widely, ranging from 33.8% to 90% for some illnesses,^15^ with the recommended optimal adherence rate being at least 80%.^16^,^17^ Consequently, there are numerous empirical studies investigating factors influencing adherence. These studies have shown that the prescribed treatment regime, patients’ overall socioeconomic status, type of disease, and prognosis all impact adherence.^3^,^18^ A more recent study by Aremu et al,^19^ and a similar study by Al Meslamani,^20^ both outlined policy recommendations for improving medication adherence, spanning the use of mass communication, advocacy for decreasing costs of medication, and the improvement of patient education.

Significant among these studies is the link between healthcare provider-patient relationships (PPRs) and adherence. Literature has repeatedly pointed to the critical role that PPRs play in health outcomes.^21–28^ In multiple studies, it has been shown that quality PPRs encourage patients to rely on and trust their physician’s advice on treatment options. One longitudinal observational study with more than 800,000 patients in Norway by Sandvik et al^27^ found that PPRs, especially long-term relationships of over 15 years, could reduce the occurrence of acute hospital admission, patient mortality, and out-of-hours care by 25–30%. Reviews have pointed to the link between treatment adherence and PPRs in numerous specific illnesses or age groups. For example, Drotar^29^ published a review from 1980 to 2008 on the association between physician behaviour and health outcomes and treatment adherence in paediatric chronic illnesses. A more recent review on diabetes management and adherence by Gow et al^30^ has pointed to the key role of support from healthcare providers and family members towards healthier behaviours and adherence.

It should be noted, however, that some research has indicated that the emphasis on the relational dimensions of healthcare have declined over the last few years as providers adopt a positivistic reductionist approach to medicine driven by scientific methods and a strong shift towards medical specialization.^23^,^31^ This is particularly true in patient-facing aspects of medicine, such as in primary care and family medicine, where relationship-based care remains a serious concern with direct implications for health outcomes.^21^ The recent COVID-19 pandemic had exacerbated the practical constraints in the healthcare space, with increased burnout rates^32^ and moral distress amongst providers that further impact the relational dimensions of care.^33^

In order to better support positive health outcomes, it is imperative that we better understand the relevant characteristics of PPRs that have direct bearings on patient adherence. With this in mind, this scoping review aims to provide an overview of contemporary knowledge on the characteristics of PPRs that are associated with treatment adherence in the past decade, and to identify where relevant gaps exist relevant for future research. The current endeavour thus expands beyond reviewing a single disease to systematically synthesize and categorize the various characteristics of PPRs that influence patient adherence identified in empirical studies.

## Methods

### Search Strategy

A scoping review methodology was chosen to explore the specific attributes of the PPR that were associated with treatment adherence, as well as discern gaps for future research. Different from a systematic review that would have allowed us to examine a certain intervention or practice within treatment adherence, and its associated quality of evidence, the scoping review ensured the examination of a wider breadth of literature across all participant ages, disease profiles, and aspects of the PPR. Our search string (see Appendix 1) was structured around the domains (1) healthcare providers (ie physicians, nurse practitioners), (2) patients (ie: in- and out-patient, or care recipients), (3) relationship characteristics (such as trust, collaboration, or communication), and (4) adherence (including cooperation and compliance). These were combined with terms for study methodologies. Each domain accounted for synonyms, spelling variations, and conceptual nuances. This string was adapted for the three databases CINAHL, Embase, and PubMed. Searches were restricted to peer-reviewed articles published between 2015–2025 to build upon prior review efforts,^34^,^35^ to present a contemporary account of the field and for relevant or actionable gaps for future research, as well as to account for resource capacities within the research team.

### Eligibility Criteria

The inclusion criteria were: (1) Studies must be peer reviewed and published between 01.01.2015 and the date of extraction, namely 07.01.2025. (2) Studies must be empirical. For example, qualitative, quantitative, or randomized controlled trials in design. (3) The included studies must directly deal with a specific, quantified aspect of the provider-patient relationship. This aspect had to be specifically named and directly related to treatment adherence. Papers were included when the link between this specific aspect of the PPR and treatment adherence makes up a significant part of the paper. (4) Papers were only included when there was a pre-existing, diagnosed, and health-related condition. Preventative measures such as lifestyle changes without a prescribed treatment or medication, vaccinations, or medical screening were excluded. (5) Papers were eligible if they reported the patient perspective, the provider perspective, or both. The complete list of included articles may be found in Table S1.

Studies were excluded if they were: (1) Non-empirical in design, such as conference papers, normative papers, or other scoping and systematic reviews. (2) Studies reporting experiences of patients suffering from mental health related conditions. (3) Reporting on the development or testing of new tools/interventions, including those that aimed to improve adherence, where no final results were reported.

### Study Selection

Following the systematic search across the three databases, results were exported and uploaded to the referencing software Covidence, whereby duplicates were automatically removed. Subsequent screening was done manually to ensure accuracy. Titles and abstracts were first individually screened by five reviewers, with each record being assessed by two reviewers. Discrepancies and uncertainties were resolved by a third reviewer.

### Data Extraction

After title and abstract screening, the full texts of potentially eligible studies were retrieved and reviewed by two authors (YJT and EH) to confirm inclusion, with each record being independently examined by both reviewers. Studies not meeting eligibility criteria were excluded with documented reasons. The research team developed a data extraction sheet capturing relevant data. This included the metadata on study design, study demographics, and attributes in the PPR (empathy, care continuity, trust, etc) which were found by the included articles to link to adherence-related outcomes. 10% of the studies were randomly selected and reviewed by a second reviewer (either AM or SM) to assess data extraction consistency and accuracy. This achieved 90% in consistency in content. Any disagreements were resolved through discussion.

### Data Synthesis

Extracted data was analysed thematically to identify key patterns in PPRs and their impact on treatment adherence. Data from a certain column (ie trust) in the extraction document on Microsoft Excel were then organized in different Microsoft Word documents (ie trust) in the PPR, within which we drew out various aspects to form sub-themes. During this process, initial themes from the extraction document were thus refined and collated. A summary table of included studies, detailing study design, population, and key findings is provided in the Appendix.

## Results

### Study Demographics

**Figure 1:**
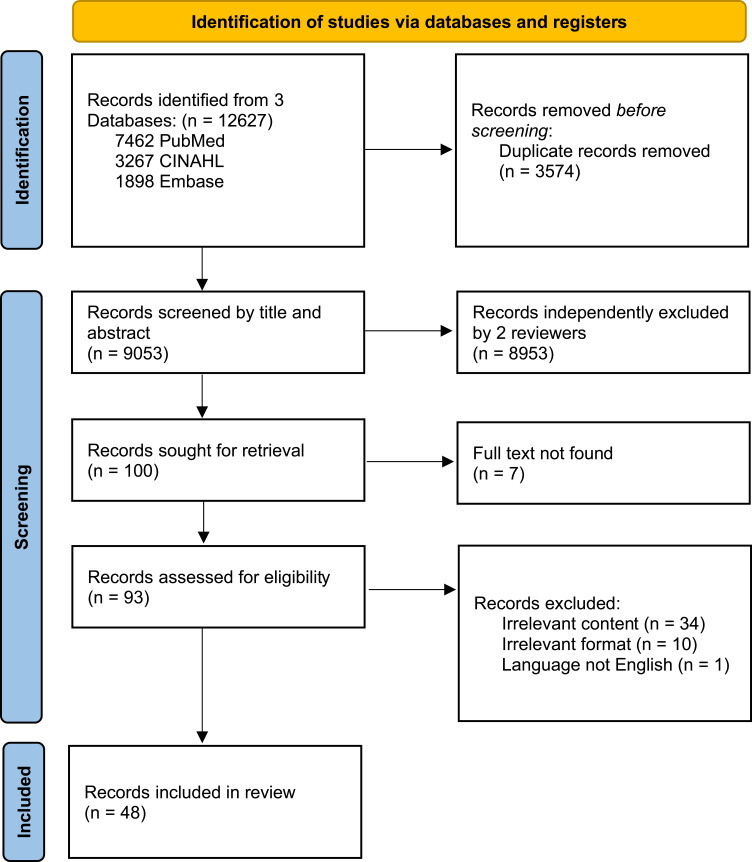
PRISMA Flow Chart.

A systematic search across three databases (PubMed, CINAHL, and Embase) revealed 12627 records; of which, 3574 duplicates were removed prior to screening, resulting in 9053 records identified for abstract and title screening. The full-text of 93 potential studies was assessed for eligibility with 45 being excluded (see PRISMA flow diagram – Figure 1 – for reasons for exclusion). Forty‑eight studies fulfil the inclusion and exclusion criteria and are relevant to the research question at hand, spanning 17,040 participants. Twenty‑three studies employed a quantitative methodology, and 17 employed qualitative, with the remaining using mixed-methods study designs (see Table 1). Included studies investigated a range of diseases, including diabetes-related (10 articles), hypertension (7), HIV/AIDS (6), cancers (5), and others (see Table 2). Thirty‑five studies (73%) studied medication adherence, 6 self-management and self-care, with the remaining studies focused on miscellaneous treatments (see Table 3). Most studies were conducted in the USA, with the remaining involving Europe, the Middle East, Asia, as well as the African continent (see Table 4). More included studies were published after 2020. Likely due to the nature of treatment adherence, almost all of the 17040 participants were patients, rather than providers (Table 5).

**Table 1:** Study Design

Study Design	Specified	Number of Studies
Quantitative	Questionnaire or survey	23
Qualitative	Interview	17
Mixed method	Interview and questionnaire	1
	Interviews and document review	1
	Observational and questionnaire	1
	Survey and interview	2
	Survey and observation	2
	Electronic monitoring data and medication journal	1

**Table 2:** Type of Disease

Type of Disease	Specification	Number of Studies
Diabetes (Total = 10)	Type 1 and 2	3
	Type 2	6
	Diabetic foot ulcers	1
Hypertension		7
Human immunodeficiency viruses (HIV/AIDS)		6
Cancer (Total = 5)	Breast	2
	Prostate	1
	Paediatric	1
	Oral	1
Multiple chronic illnesses		3
Rheumatoid arthritis		2
Lupus		2
Actinic Keratosis		1
ADHD		1
Asthma		1
Atopic Dermatitis		1
Chronic Kidney Disease		1
Cystic Fibrosis		1
Dyslipidemia		1
Fibromyalgia		1
Hemodialysis or Peritoneal Dialysis		1
Kidney Transplants		1
Multiple Sclerosis		1
Cardiovascular diseases		1
Chronic Diseases		1

**Table 3:** Treatment

Treatment	Specified	Number of Studies
Medication (Total = 35)	–	27
	Antiretroviral therapy	4
	Adjuvant endocrine therapy	2
	Chemotherapy	2
Self-management (Total = 6)	-	4
	Limiting weight-bearing activity	1
	Regimes (dietary)	1
Medication and self-management		2
Medication, diet, exercise		1
Regimes (medical)		1
Topical field-directed therapy		1
Active surveillance, prostatectomy or external beam radiation		1
Not specified		1

**Table 4:** Country of Study

Continent	Country	Number of Studies
North America	USA	24
Europe (Total = 9)	Austria	1
	France	2
	Italy	1
	Poland	1
	Switzerland	1
	UK	3
Middle East (Total = 6)	Iran	2
	Israel	1
	Lebanon	1
	Pakistan	1
	Saudi Arabia	1
Asia (Total = 5)	Bangladesh	1
	Japan	1
	Korea	1
	Singapore	1
	Vietnam	1
Australia	New Zealand	1
Africa	Kenya	1
Multiple continents		2

**Table 5:** Number of Participants

Metric	Specified	Number of Participants
Type of Participants	Patients	16,933
	Providers	107
Mean Participants per study		355
Median Participants per study		142

### Overview of Themes

Communication was by far the most prevalent theme, mentioned in 33 of the 48 articles (see Table 6). Thereafter, collaborative decision-making and trust were both mentioned in 19 articles. This was followed by empathy, which was noted in 8 studies, and care continuity, which appeared in 7 studies. Finally, discrepancies between providers and patients were explicitly examined in 3 studies.

**Table 6:** Overview of All Attributes and Number of Studies

Theme	Number of Studies (Total: 48)
1. Communication	33
2. Collaborative decision making	19
3. Trust	19
4. Empathy	8
5. Care continuity	7
6. Discrepancies between providers and patients	3

#### Attribute 1: Communication

Communication was by far the most mentioned attribute of PPRs throughout the included studies with 33 noting some dimension of communication, such as information delivery; style of communication; or style of engagement, that correlated with treatment adherence. Information delivery that was comprehensive and clear improved adherence. Patients also valued communication that is honest, simple, educational, and tailored to their individual needs. Providers that engaged with their patients in a respectful, empowering, attentive, and proactive way also positively influenced adherence rates. Ultimately, good communication fosters a relationship where patients feel comfortable seeking help and discussing difficulties openly.

Tailoring advice according to patient needs and unique circumstances was significantly associated with better PPRs.^36^,^37^ Providing comprehensive information^38−46^ with high clarity and consistency,^36^,^45^,^47–50^ leaving ample opportunities for patients to ask questions and receive answers,^40^,^41^,^51^ and confirming patient understanding^39^ were important factors in improving provider-patient communication. As adherence was linked to whether patients believed the medication to be necessary, the included studies noted that providers should supply information that underlines necessity, even if patients were already seeing the effects of the medication.^52^ Another study from Amin et al^39^ found patients to be 3.23 times more likely to adhere to anti-hypertension medication when they have more positive exchanges of information. Conversely, when young adults transitioning to adult renal care felt their questions were unanswered and that they were not provided with enough information on patient expectations, they were more likely to feel unsafe and distrustful to the point of even dropping out of care.^41^ Regarding treatment recommendations, the included studies demonstrated that providers can improve communication by addressing patient concerns and fears sufficiently,^40^,^43^,^53^ as well as explaining their reasoning behind recommendations.^36^,^47^ Likewise, information delivery should also extend to the provision of written materials, as the lack thereof can cause frustration, feelings of disrespect, anger, and distrust toward providers.^41^ Communication that was honest, simple, and educational was linked to adherence.^36^,^37^,^41^,^46^,^47^,^52–55^

Communicating in a respectful manner, rather than condescending or paternalistic, was important for adherence to provider recommendations.^36^,^40^,^44^,^50^,^56^ Words such as “must” or using excessive repetition did not result in improved motivation for adherence. Studies demonstrated that patients wished to be respected for their individuality and unique requirements.^36^,^54^ One study by Soyoon and Ekaterina^57^ on diabetes self-care noted the positive effects on adherence of providers who were interested to hear about patient’s personal beliefs and difficulties, and who were empowering in their communication style. One study by Anderson et al^58^ on the sexual health challenges for Black women with early-stage breast cancer noted the biased beliefs that may be present in male physicians as contributing negatively to the quality of the PPR. Providers who were more willing to communicate^59^ and provided enough time for patients had a greater impact on adherence.^39^,^41^,^51–53^,^55^,^60^ Engaged providers who were attentive and not distracted, as well as proactive and respectful, enhanced adherence.^40^,^45^,^50^,^53^,^55^ Furthermore, providers who were caring and considerate further facilitated adherence.^40^,^41^,^45^,^54^ In the study by Anderson et al^58^ on Black women’s experiences of early-stage breast cancer, the authors found that clinicians who were female and affirming were more likely to improve adherence to adjuvant endocrine therapy. Affirmation was confirmed as an adherence promoting aspect by other studies,^36^,^47^,^53^ as well as providers who acknowledged and celebrated adherence, or provided emotional support and encouragements.^36^,^44^,^50^,^52–54^ Continued engagement by providers who followed up with patients and provided accountability checks was further linked with positive adherence.^53^,^54^

Providers’ personality was important, especially when it was relevant to enhancing communication skills. Having good interpersonal skills and the ability to communicate or listen was appreciated by patients.^40–42^,^50^,^54^,^60^,^61^ Having meaningful and high quality communication and discussions was identified by three studies to improve adherence.^37^,^43^,^62^

Overall, good communication left patients feeling more comfortable in seeking help, reaching out to their providers,^40^,^45^ or staying open to discussions.^36^,^47^,^63^ Studies noted that patients should not be blamed for non-compliance as this risks them becoming reluctant to receive further advice.^47^,^64^ One study by Fernandez et al,^53^ which examined the reception of HI care in low-income minority women in South Florida, found positive effects from both reassurance and comfort providing. This was especially relevant when patients were having a difficult time adjusting to a disease or adhering to their treatment. Providers who offered support both administratively and emotionally supported patients to maintain treatment. This support included acquiring medications and providing digital reminders via text-message to take medications. Such support was seen by providers as a way of giving holistic care and letting patients feel comfortable talking about their difficulties to adherence, rather than disregarding patients before their concerns could be adequately addressed.

In addition to the efforts of patients or providers themselves, wider family members may also contribute to improved communication within the PPR. In a USA study by Jamil et al^63^ on medical adherence and health beliefs of South Asian immigrants with diabetes, the authors found that family members could assist adherence to medication and facilitate communication with providers.

As the most mentioned attribute in our review, we found that engaging in tailored guidance with patients frequently, clearly, respectfully, and proactively meaningfully improved adherence. This is because such communication allowed patients and their families to feel more comfortable to seek care, ask questions about their treatment, and feel more reassured that they were properly informed about their care.

#### Attribute 2: Shared Decision-Making

Our review suggests that shifting from traditional paternalistic models of care toward more shared decision-making (SDM) is a promising strategy for improving treatment adherence. In our review, six studies noted a preference from both providers and patients for more patient-centred approaches,^41^,^45^,^49^,^50^,^56^,^64^ and nine additional studies noted the positive role of greater patient involvement for adherence.^36^,^40^,^51^,^57^,^59^,^63^,^65–67^ For example, in an Austrian study by Beichler et al^59^ on patients living in HIV and receiving antiretroviral therapy, the authors found that patients who were more adherent also had more involvement in their own care. This involvement consisted of participating widely in discussions with their providers. Furthermore, studies concluded that patients should bear greater responsibility to improving adherence,^65^ while providers should work to sufficiently incorporate their patients’ preferences for treatment,^40^,^56^ individualize and tailor treatment regimens,^47^ or proactively review lab-work with patients.^53^ Interestingly, one article that reported no relationship between having a collaborative decision-making model and adherence^68^ advocated for greater self-management behaviours from the patients. Overall, a shared decision-making model and greater patient-involvement contributed to better adherence.

#### Attribute 3: Trust

Trust in healthcare providers emerged as a significant factor influencing patient adherence to treatment, with eleven studies confirming that trusted providers improved adherence.^43^,^47^,^61^,^66^,^69–75^ Trust and confidence in physicians led patients to believe in the necessity and benefit of their medication or therapy,^38^,^42^,^43^,^69^,^76^ for patients to continue medication despite negative side effects,^40^ improved patient’s acceptance of information given about their treatment,^69^ and overall allowed more positive discussions around diseases.^52^ What constitutes, or affects, such trust in providers was elaborated in a limited number of studies. Being friendly and respectful,^52^ welcoming and supportive,^41^,^42^ making the effort to learn the patient’s narrative,^53^ prioritizing patient wishes despite lack of clinical validity,^77^ and giving explicit clarifications on care expectations^41^,^42^ were all noted to build trust.

Conversely, mistrust was associated with non-adherence. For example, a study on ART adherence among 458 HIV-positive African-Americans found the negative impact of race-based medical mistrust on adherence levels.^76^ Here, patients completed the Group-Based Medical Distrust Scale, in which one question included, “People of my race cannot trust doctors and healthcare workers”. This question isolated the influence of discrimination in the trust parameter. Although neither general medical mistrust nor trust in providers were found to significantly predict medication adherence, higher levels of race-based medical mistrust did predict lower medical adherence, medication necessity and concern beliefs. These results were confirmed by another study by Salt et al^74^ on medication adherence within rheumatoid arthritis sufferers. The authors found that white participants (compared to Hispanic participants) were more likely to trust their providers, perceive higher quality of communication, be satisfied, and were more adherent to prescribed medication.

In addition, two studies found no correlation between mistrust and adherence.^73^,^76^ One of which, by Pellowski et al,^76^ isolated the factor of race-based mistrust that, once removed, did not link adherence to general mistrust. Another study on trust beliefs held by children with asthma and their mothers, by Rotenberg and Petrocchi,^73^ found that mother’s trust beliefs neither correlated with adherence nor with their own childrens’ trust beliefs. The authors reflected that this result ran counter to the idea of socialization whereby mothers influence their children’s beliefs.

To sum up, some evidence suggests that trust in the provider contributed to treatment adherence, in terms of patient’s confidence in their prescribed treatment and the necessity to continue against side-effects.

#### Attribute 4: Empathy

Empathy was a concept that encompassed many distinct aspects of the PPR in our review, including comfort to share information,^58^ respect,^39^,^53^ taking the patient’s perspective,^77^ feeling safe,^41^ or validated in the PPR.^36^,^43^ Empathy itself was measured statistically as a cohesive concept by authors that contributes to improved adherence.^78^,^79^ This included listening to patients^43^ or praying with patients before therapies.^77^ Overall, empathy was a relatively ambiguous term that warranted a clear definition in many articles and currently includes many relational aspects that all contributed to adherence.

#### Attribute 5: Care Continuity

A consistent and long-term provider-patient relationship was positively associated with improved adherence.^52^ Three studies noted that care may be interrupted by changing providers.^41^,^47^,^54^ A study by Kielhold et al^54^ found that patients were reluctant to relocate due to the fear of disrupting their existing PPRs. Studies also indicate that continuity of care was related to favourable emotions towards prescribed treatment^36^ and a greater willingness to speak more openly about sensitive health information.^58^ Noteworthy is that a lengthier PPR appeared to decrease the preference for SDM. This may be due to familiarity with a provider that leads to increased trust.^67^ To sum up, having a longer relationship with the provider contributed to positive treatment adherence.

#### Attribute 6: Discrepancies Between Providers and Patients

Three studies explicitly examined noteworthy discrepancies between perspectives from providers and patients.^37^,^75^,^80^ Schlegel and Leray^75^ noted differing views of non-compliance; with patients seeing non-compliance as rational, while providers labelled it as deviant. Their study highlighted how providers’ willingness to involve patients and validate patient autonomy improved adherence, underscoring the role of trust. Two further studies by Eaton et al (2020) and Hunter et al (2023) considered barriers to compliance, with both mentioning that barriers were perceived and conceptualized differently by providers and patients. Their studies suggest that such discrepancies may exacerbate non-compliance. They recommended improving open, patient-centred communication and trust to help overcome these barriers.

### Confounders

Certain confounders were noted to affect the contribution of the PPR to adherence. These included: Gender, with ambiguous results denoting which gender may improve or hinder adherence.^42^,^59^,^64^,^81^ For example, two studies noted that women patients were less adherent than men, as they were more likely to believe that providers were overprescribing medications, ie lower medical necessity.^42^,^81^ Greater health literacy and knowledge regarding the specific disease contributed to better adherence.^37^,^45^,^52^,^59^,^64^,^69^,^71^,^80^ Nevertheless, the education level of the patient did not automatically lead to unilaterally improved adherence.^37^,^42^,^52^ Studies mentioned age as a confounder, but did not form a consensus on its impacts on adherence.^52^,^59^,^72^ Religious beliefs,^45^,^63^,^64^ such as in the study by Ansari et al,^64^ where patients believed in the positive impacts of religion (God’s will and destiny) to cure the disease, rather than medication or treatment prescribed. Closely linked were beliefs in alternative medicine rather than prescribed therapy,^52^,^63^,^64^ or disagreements with treatment plans,^45^,^64^ influencing adherence.

Practical considerations impacted adherence levels. This included: Financial difficulties,^37^,^52^,^71^,^81^ living further away from medical facilities,^52^ and inconvenient medical transportation.^80^ Patients with greater self-efficacy,^36^,^65^,^82^ higher coping abilities,^54^,^56^,^65^,^68^ higher conscientiousness,^45^,^52^ less psychologically reactive or more patient,^72^ and taking more responsibility of their own conditions^41^ had greater adherence. Those fearful of injections,^47^,^75^ fatigue with regimens,^75^ physical fatigue in general,^80^ as well as having previous negative side effects from treatment,^37^,^52^,^71^ all contributed to decreased adherence. Conversely, lower medical dosage or previous positive experiences with medication were associated with adherent patients. Disease burden was a key determinant to the decrease of adherence, along with the complexity of chronic diseases^45^,^56^ as well as other social obligations.^36^,^56^,^80^ Having a worsening health condition was noted to improve motivation to take care of one’s health and adjust to new medical regiments.^45^,^63^,^66^,^79^

## Discussion

This scoping review has uncovered the complex, yet critical role PPRs play in treatment-adherence. A review of the literature confirms what is widely held to be true: The necessity of providing personalized, patient-centred care that integrates cultural, socioeconomic, and experiential factors to improve treatment adherence, particularly among marginalized groups such as Black women with breast cancer, South Asian immigrants, or people living with HIV/AIDS. Emphasis in the identified studies was placed on enhancing provider competencies in interpersonal communication, empathy, and shared decision-making to strengthen therapeutic alliances and foster mutual understanding.

In particular, the empirical literature demonstrates the importance of relational aspects of medicine for adherence. That is to say, the literature establishes PPRs as having a direct impact on patient adherence. A survey of the literature indicates five important attributes of the PPRs that promote adherence, namely: Communication, shared decision-making, trust, empathy and the temporal duration of the care relationship. Of these, communication was by far the most noted attribute across all 48 included studies. This attribute of the PPR encompasses the amount or type of information delivered during consultations, the engagement style, and the consistency of support, all of which were empirically established as being crucial for patient adherence. This was supported by the attribute of shared decision-making which affirms the impact of recent shifts within the medical profession. As Vrijens and colleagues^83^ on the taxonomy of adherence has demonstrated, the medical profession has been marked by a recent move away from the notion of “compliance” (the expectation that patients will uncritically follow a physician’s recommendations) toward the notion of “adherence” (the active agreement by patients engage with treatment). This shift reflects a broader, patient-centered movement within healthcare over the last few decades. Patients are increasingly understood to be more than passive bystanders in their treatment. They are active partners who inform and actively choose their care.^2^,^3^,^84^

That adherence is causally linked to PPRs, must however, be considered in the context of two other significant shifts in medicine. First, as Rudebeck^31^ has argued, the drive toward medical specialisation fuelled by the adoption of a more positivistic, reductionist approach to clinical care by providers increasingly focuses medicine away from its human dimension. That is to say, as providers increasingly hone their skills within a narrow competency, their focus shifts from whole-patient care toward a narrow focus. This risks providers’ losing sight of critical aspects of healthcare, such as the relationship between PPR and adherence. This process of increasing specialisation may find tailwind in the context of a second significant trend that is rapidly taking place in the field of medicine: Digitilisation.

The societal trend toward the incorporation of digital technologies such as artificial intelligence (AI) have gained increasing attention in medical fields such as elder care and personalized medicine. Although we did not include the use of technologies in our review, we note greater uses in recent studies of digital application or smart medication dispensers to measure medication adherence, or activity monitors for lifestyle changes.^85–87^ These tools may improve communication, care delivery, and empower patients to improve autonomy.^88^ The collection of more health data could, in addition, enhance information needed in consultations for providers to understand more about issues of non-adherence. Policy recommendations from Al Meslamani^20^ also encourage government-support initiatives and healthcare providers to adopt or fund digital tools in improving adherence. Such technologies may be especially helpful for patients with less physical access to healthcare providers, or those that may not be available to seek care frequently enough. The use of telehealth, or digital consultations here may indeed support health outcomes.

Nevertheless, technologies risk exacerbating the digital divide for patients that are uncomfortable with managing their conditions using them, such as those with less digital literacy. In addition, as our review has highlighted that the need for maintaining a PPR with communication, trust, empathy, and care continuity may be disrupted by technologies replacing in-person consultations. It is unknown, and worthy of exploration, how to weigh the benefits of technologies to improve health outcomes, with the replacement (entirely or in part) of PPRs by AI conversation agents. In light of our review, this presents a serious risk to both patient adherence and consequently general health outcomes.

Finally, a significant gap in the current state of research exists around defining and operationalising critical attributes of the PPR for adherence. While the empirical research overwhelmingly affirms attributes such as communication, shared decision-making, trust, empathy and care continuity as significant attributes of the PPR, what these attributes concretely entail, and the mechanisms through which they influence adherence, are insufficiently specified. None of the studies included in our review offered explicit definitions or measurement strategies for constructs such as “communication”, “trust”, or “empathy”. For instance, no study provided either a standardised conceptualisation of communication nor articulated a detailed working definition. Consequently, studies purporting to measure “communication” encompassed highly heterogeneous phenomena, often without visible critical reflection, implicitly assuming their own conceptualisations to be self-evident and widely accepted. Some studies focused on the informational content conveyed by providers, others on communicative style, or the level of patient-provider interactivity. This conceptual heterogeneity precludes the development of an empirically rigorous account of which specific communicative attributes meaningfully contribute to adherence-affirming PPRs. This conceptual fragility extended to other attributes of PPRs identified in our review, namely trust, empathy, or shared decision-making. Across studies, these constructs were repeatedly insufficiently defined and, consequently, measured in markedly heterogeneous ways. It is, therefore, difficult to ascertain what is actually meant when one says provider empathy enhances adherence, or that effective communication can support adherence-affirming PPRs. This remains a substantial limitation in the current state of art regarding our understanding of the mechanisms through which PPRs affirm adherence.

### Strengths and Limitations

The strengths of this review may be outlined in two dimensions: Breadth of scope and specificity in relational aspects. The inclusion criteria were designed to cut across any disease or illness and any single aspect of the PPR, which allows subsequent research to holistically build upon PPR as a factor of adherence. The wider scope also fulfils our aims as a scoping review to capture the breadth of the field and the rich empirical work that has been done thus far. At the same time, our efforts also categorized the specific relational attributes within the PPR that could serve to compare and contrast their impacts on adherence. We were hence able to draw out that communication was comparatively critical amongst other relational attributes, which warrants greater attention in future guidelines and research.

The results of this scoping review are also subject to limitations. First, as noted in our discussions above, the lack of consistent definitions for key constructs – such as communication, empathy, shared decision-making, and trust – across the included studies makes it difficult to accurately reflect the relative importance of these PPR attributes for adherence. The conceptual ambiguity and inconsistent operationalization of these attributes make it difficult to compare findings, synthesize mechanisms, or draw fine-grained conclusions about which relational components truly influence adherence. This issue directly constrained the interpretive strength of the review. Second, feasibility necessitated a focus on non-mental health related conditions. This focus limits the generalizability of the findings to non-mental health conditions only. It is possible that adherence within psychiatric or psychological care may involve additional mechanisms not captured here. Third, the methodological variability of the included studies. The included studies varied substantially in design (qualitative, quantitative, cross-sectional, mixed-methods), measurement quality, and adherence metrics. Since most studies relied on self-report measures, which are susceptible to recall bias and social desirability, true adherence may be overestimated. In addition, it should be noted that there may exist publication bias in favour of studies reporting positive relational effects. This could potentially inflate the apparent strength of associations between adherence and the distinctive attributes of the PPR reported here. Fourth, limited provider perspectives. Most included studies centred on patient reports; relatively few incorporated provider perspectives (107 vs 16,933 participants). This asymmetry constrains the insight into relational dynamics from both sides of the therapeutic encounter. Furthermore, the predominance of US-based studies may bias findings toward Western healthcare norms and limit applicability to systems with different cultural expectations of PPRs. Finally, as a scoping review, this study aimed to map the literature comprehensively rather than evaluate the quality or risk of bias of included studies. Consequently, the strength of conclusions is limited by the methodological robustness of the primary research.

## Conclusion

The scoping review of 48 peer-reviewed empirical studies published between 2015 and 2025 has demonstrated that the relational dimensions of health care play a critical role in supporting treatment adherence across a broad range of health conditions. We found communication, shared decision-making, trust, empathy, continuity of care, and the lack of discrepancy in the PPR were positively associated with treatment adherence. Communication emerged as the most consistently influential factor, underpinning the importance of clear information exchange, patient engagement, and provider responsiveness. However, the review also identified significant conceptual and methodological gaps in how relational aspects are defined and measured. Future research should employ clearly defined and systematically measured notions of communication, trust, empathy, and shared decision-making if empirically grounded strategies for fostering adherence-affirming PPRs are to be meaningfully advanced. For practically translating these findings to improve treatment adherence, we suggest that greater emphasis should be placed on developing clear, well-defined steps for providers in the clinical or care setting to improve the frequency, quality, and activeness of the engagement with their patients. These steps should proactively allow any discrepancies, doubts, and challenges relevant to the treatment regime to be addressed prior to affecting adherence. In this process, the conceptual gaps of the relational dimensions in the PPR should be correspondingly clarified.
